# Evaluating Treatment Adherence in Children and Adolescents with Type 1 Diabetes: The Impact of the Adherence Starts with Knowledge-12 Score on Metabolic Control

**DOI:** 10.3390/children12040463

**Published:** 2025-04-03

**Authors:** Semine Ozdemir Dilek, Fatma Özgüç Çömlek

**Affiliations:** 1 Department of Pediatric Endocrinology, Adana City Training and Research Hospital, Adana 01370, Turkey; 2 Department of Pediatric Endocrinology, Medical Faculty, Selçuk University, Konya 42250, Turkey; fatma.ozguc@selcuk.edu.tr

**Keywords:** type 1 diabetes, Adherence Starts with Knowledge-12, treatment adherence, metabolic control

## Abstract

**Background/Objectives:** This study sought to identify key barriers to treatment adherence in children and adolescents with type 1 diabetes (T1D) using the Adherence Starts with Knowledge-12 (ASK-12) questionnaire and to evaluate its impact on metabolic control, providing insights for optimizing T1D management. **Methods:** A total of 160 children and adolescents with T1D aged 5–18 years who sought treatment from a pediatric endocrinology outpatient clinic between June and August in 2022 were prospectively examined. The patients’ low treatment adherence (LTA) or high treatment adherence (HTA) was determined based on their ASK-12 questionnaire scores. Two pediatric endocrinologists reviewed the participants’ medical records and then classified them into two groups: tight metabolic control and poor metabolic control. **Results:** LTA, which was determined based on the participants’ ASK-12 scores, was significantly associated with puberty, presence of diabetic ketoacidosis, and daily self-management (*p* < 0.001, *p* < 0.001, and *p* < 0.001, respectively). Those whose ASK-12 scores indicated LTA were older and had a longer duration of T1D, higher hemoglobin A1c levels, and lower BMI-SDS values than those with HTA) (*p* < 0.001, *p* < 0.001, *p* < 0.001, and *p* < 0.001, respectively). A total of 94 (59%) participants were indicated to have HTA, but 24 (25.5%) of them were found by the clinicians to have poor metabolic control. **Conclusions:** The ASK-12 questionnaire scores can identify pediatric patients with T1D who exhibit LTA and thus may be beneficial for early recognition of low adherence. Approximately 25% of the patients with ASK-12 scores indicating HTA were at risk of poor metabolic control. Puberty, duration of T1D, BMI-SDS, HbA1C, and parental involvement alongside ASK-12 score may be considered to improve treatment compliance. Integrating these variables into adherence assessments may enhance treatment compliance and improve long-term outcomes in pediatric T1D management.

## 1. Introduction

Childhood type 1 diabetes (T1D) is a chronic disease resulting from autoimmune damage to pancreatic β cells, and it necessitates insulin therapy [1]. Approximately 1.2 million children and adolescents worldwide have T1D [2]. The prevalence of childhood T1D has increased in recent years, with peak incidences occurring in children aged 4–6 and 10–14 years [3].

A significant concern with T1D is that it requires lifelong insulin therapy, especially because no preventive treatment has ever been approved [4]. Additionally, children with T1D and their parents may experience anxiety and burnout due to the psychological burden resulting from knowing that this disease is a lifelong clinical condition, and due to other concerns, such as puberty and lifestyle changes [5]. In children and adolescents with T1D, tight metabolic control with insulin therapy is associated with low hemoglobin A1c (HbA1c) levels and reduced macrovascular and microvascular complications [6]. Therefore, compliance with insulin therapy is critical to achieving optimal glycemic control [7].

Treatment adherence refers to the degree to which patients follow a recommended treatment regimen. It is an important aspect of patient care and an important step in reaching clinicians’ objectives [8]. The concordance between patients’ and physicians’ assessment of medication adherence is crucial in T1D management and in accurate identification of non-adherent patients. For accurate identification of medication adherence-related problems and for improving communication between physicians and patients, the Adherence Starts with Knowledge-20 (ASK-20) questionnaire was improved [9]. A shorter version of the ASK-20 questionnaire (i.e., the ASK-12) is currently being used [10].

Factors including compliance and self-management and patients’ knowledge of managing diabetes-related emergencies must be studied to understand their impact on the physical and psychological health of patients with T1D. Using the ASK-12 questionnaire score, we aimed to identify patients who exhibited non-adherence and to detect serious adherence-related issues in patients with T1D.

## 2. Materials and Methods

In this study, 160 patients with T1D who were aged 5–18 years and were followed-up in an outpatient clinic between June and August in 2022 were prospectively examined. Exclusion criteria included patients with insufficient data and those who did not apply for regular outpatient clinic visits. All patients and their parents agreed to participate in the study, which was approved by the Medical Clinical Research Ethics Committee (2022/354) and conducted in accordance with the Declaration of Helsinki.

After the ASK-12 questionnaire was administered to the participants, they had regular check-ups with two pediatric endocrinologists at least three months apart for at least six months. The criteria set by the International Paediatric and Adolescent Society (ISPAD) in 2022 were used in this study to assess the onset of T1D, as well as the medical records of children with diabetic ketoacidosis (DKA) (i.e., plasma glucose > 200 mg/dL). To test for ketonemia, blood β-hydroxybutyrate (βHB) levels were measured using the FreeStyle Optium β-Ketone Test Strips (Abbott Diabetes Care Ltd., Witney, UK) in conjunction with the FreeStyle Optium Neo meter (Abbott Diabetes Care Ltd., Witney, UK). A 1.5 µL capillary whole-blood sample was applied to the test strip. For ketonuria, the dipstick method for urine was used (URIT 11G test strips, URIT^®^, Changchun, China). Those who were positive (venous pH < 7.3 or bicarbonate < 18 mmol/L) and those without DKA (i.e., glucose > 200 mg/dL, ketonemia- or ketonuria-negative, and venous pH > 7.3 or bicarbonate > 15 mmol/L) were determined (Radiometer ABL Go Flex device SP90 XL, 944-457, Radiometer Medical ApS, Copenhagen, Denmark). We used the potentiometric glass electrode method for pH measurement and the application of the Henderson–Hasselbalch equation for the calculation of HCO_3_^−^ [11]. The patient’s emergency service visits due to one or more DKA episodes within one year were noted. The partial remission (PR) criteria was definied as IDAA1c (HbA1c (%) + 4 * daily insulin dose) ≤ 9 [12]. All participants received an insulin regimen comprising once-daily fixed-time long-acting glargine and rapid-acting insulin analogues (e.g., insulin lispro, aspart) administered at each main meal. Dosages were individualized based on the patient’s total daily insulin needs, carbohydrate intake, and pre-meal glucose levels, with adjustments guided by self-monitored blood glucose (SMBG) and ISPAD guidelines glycemic targets [13]. Additionally, all patients were prescribed glucagon for emergency management of severe hypoglycemia.

Height, weight, and body mass index standard deviation (BMI-SD) scores were evaluated and adjusted for Turkish children according to age and gender [14]. Two pediatric endocrinologists examined the participants’ medical records and then classified them into two groups: tight metabolic control and poor metabolic control. The participants’ data were collected over a 14-day period using self-monitoring fingerstick blood glucose ≥ eight times a day and recorded on on charts and the findings were analyzed. In the routine follow-up of patients with T1D, fingerstick capillary blood glucose measurements via glucometer data (over the preceding two weeks) and the accuracy of the blood glucose measurement chart were assessed. HbA1c levels was measured using high-performance liquid chromatography (HPLC) on the Trinity Biotech Premier Hb9210™ system (Bray, Ireland/Kansas City, MO, USA) and tight metabolic control was achieved by considering the values of HbA1c below 7% and blood glucose monitoring charts > 70% of the time in range (TIR; % time with glucose 70–180 mg/dL) [13]. The assessment of pubertal stages was conducted using the Tanner stages; testicular volume was measured with an orchidometer, and breast development was evaluated through visual inspection and palpation [15]. The onset of puberty was indicated by a testicular volume of ≥4 mL in boys and by breast development (≥Tanner stage 2) in girls.

The patients could read and understand Turkish and could accomplish a ten-point numeric rating scale (NRS). Informed written consent was obtained from all participants, and approval was sought from participants aged > 8 years. The parents of 18 participants aged < 8 years made the survey notification and completed the survey questionnaire. The survey questionnaire was designed to be easily understood by children and their parents. The Adherence Starts with Knowledge (ASK-12) questionnaire was carefully translated into Turkish to retain its original meaning; the Turkish version was translated back into English and then compared with the original version. The Turkish-adapted ASK-12 treatment-adherence-scale test was then administered to the participants. The ASK-12 is based on ASK-20 and has been confirmed to have sufficient internal consistency and test–retest reliability [10].

A self-report questionnaire, ASK-12 is a 12-item scale consisting of three adherence-related subscales: behavior (5 items), health beliefs (4 items), and inconvenience/forgetfulness (3 items). The health belief and inconvenience subscales include five response options: strongly agree, agree, neutral, disagree, and strongly disagree. The total score ranges from 12 to 60. The classification of high and low adherence based on ASK-12 score thresholds (>22 for low treatment adherence [LTA] and <22 for high treatment adherence [HTA]) was derived from previous studies in adult and pediatric populations [16,17]. The involvement of parents in daily glucose monitoring, hypoglycemia and hyperglycemia management, and daily insulin therapy is denoted herein as parental management (PM), whereas the lack of parental support is denoted as patient self-management (PSM).

### Statistics

The SPSS (Statistical Package for the Social Sciences) 25.0 program was used for statistical analysis. Categorical measurements are summarized as numbers and percentages, whereas continuous measurements are presented as means and standard deviations (median and minimum–maximum, when necessary). Chi-square and Fisher’s exact tests were used to compare categorical values. The normal distribution of data was assessed using the Shapiro–Wilk test. Parameters that did not show normal distribution were analyzed using the Mann–Whitney U test. Statistical significance was set at 0.05 in all tests.

## 3. Results

This study included 160 participants (96 female and 64 male). The participants’ mean age was 11.7 ± 3.3 years, and the mean age at disease onset was 8.1 ± 2.3 years. Physical examination showed that 90 (56.3%) children had entered puberty. Meanwhile, 26 (16.3%) participants were in PR. Per the clinicians’ assessments, 80 (50%) had poor metabolic control and 82 (51.2%) had poor dietary compliance (Table 1). A total of 36 (22.5%) participants exhibited diseases accompanying diabetes, including diabetic nephropathy (12 patients), celiac disease (4 patients), and Hashimoto thyroiditis (20 patients).

A total of 66 participants who exhibited LTA based on their ASK-12 scores were examined, and 50 (75%) considered refusing insulin treatment, had terminated the treatment, or had no insulin or glucagon on hand. However, they stated that there had been no problems reaching the diabetes team whenever a need for an insulin or glucagon dose arose. LTA, as indicated by ASK-12 scores, was found to be significantly associated with puberty, frequency of DKA, and daily PSM (*p* < 0.001, *p* < 0.001, and *p* < 0.001, respectively). Patients with ASK-12 scores indicating LTA were older and had a longer duration of T1D, higher HbA1c levels, and higher annual DKA frequency (*p* < 0.001, *p* < 0.001, *p* < 0.001, and *p* < 0.001, respectively) as well as lower BMI-SDS values than those with ASK-12 scores indicating HTA (*p* < 0.001).

Those with ASK-12 scores indicating HTA had a high frequency of PR, diet compliance, and tight metabolic control (*p* < 0.001, *p* < 0.001, and *p* < 0.001, respectively). Based on the ASK-12 score, 94 (59%) participants exhibit HTA, but 24 (25.5%) of them were found to have poor metabolic control (Table 2).

## 4. Discussion

This study investigated the treatment adherence of children and adolescents with T1D based on self-reports. It compared the pediatric endocrinologists’ evaluation with the participants’ state of metabolic control and medication adherence to determine the difficulties experienced by the participants during the follow-up period. Treatment adherence in pediatric T1D cases significantly impacts their prognosis, such as improved glycemic control, lower acute complications (i.e., reduced DKA, hypoglycemia), and long-term effects (growth pattern, reduced microvascular and macrovascular complications), and of quality of life [6,7,18,19].

We found that longer duration of T1D was associated with LTA. Of the patients who exhibited LTA, 81.8% have entered puberty. According to the study, puberty is an influencing factor for LTA. T1D management during puberty is indeed challenging. Adolescents’ tendency to experiment with hazardous behavioral patterns in disease management to gain independence and develop an individual identity may result in compliance issues in diabetes administration [20]. In addition, depression and anxiety have been reported to be more prevalent in adolescents with T1D than in the general population [21]. According to Rechenberg et al. [22], anxiety daily stressors like academic or extracurricular activities may affect and limit liabilities involved in T1D management. Diabetes-specific eating disorders and attendant psychopathologies are also common in children and adolescents with T1D [23]. Additionally, having a chronic disease and knowing that it will persist affect treatment compliance by increasing the feeling of burnout amongst patients [5]. Recently, a personalized approach has been important in improving the management of T1D in adolescents; psychosocial support, repeated diabetes education, family involvement, and targeted interventions to increase treatment adherence are also emphasized [24].

Daily PSM during the course of treatment was also associated with LTA, which was based on the ASK-12 scores of the participants with T1D. While the self-management ability and engagement of children and adolescents with T1D patients are obligatory, the collaboration of supportive parental involvement and shared responsibility is crucial for effective management in T1D care. Several factors may be considered when determining the degree of self-management and parental contribution in T1D management: psychosocial maturation, patient awareness, and ability to participate in follow-up and intervention in the face of chronic disease [25,26]. In the Family Management of Diabetes clinical trial, Marker et al. [26] found that the parental involvement and low conflict groups exhibited high treatment adherence, optimal glycemic control, and psychosocial accordance. A study in Spain of pediatric T1D found that joint parental supervision of diabetes care was associated with lower HbA1c and higher adherence, emphasizing the role of family structure in T1D management [27]. Findings similar to ours have shown that decreased parental involvement in T1D management improved glycemic outcomes initially, but it subsequently elevated HbA1c levels [28]. In cases of longer duration of T1D and when patients have entered puberty, evaluating patients’ knowledge and treatment compliance is essential during follow-ups. Early independence during puberty and reduced parental involvement might diminish treatment compliance of patients with T1D, leading to poor metabolic control.

Pediatric patients with T1D are in a permanent state of anxiety about taking responsibility due to the recommended daily routines and the need for consistency; this persistent state may be a contributing factor for suboptimal glycemic control [18]. According to Sohayla et al. [29], adolescents with T1D who monitored their blood or interstitial glucose levels at least four times a day had lower HbA1c levels; in contrast, 97% of patients with non-adherence to glucose monitoring did not reach tight metabolic control. In addition, integrating the Self-Report Behavioral Automaticity Index with T1D has enhanced adolescents’ ability to complete tasks and improve self-management skills, ultimately leading to recommended glucose levels [30]. Herzer et al. [19] found that anxiety is associated with poor metabolic control and increased HbA1c levels; their study of treatment compliance showed that anxiety is a persistent stress factor for adolescents with T1D. Moreover, our results suggest that low BMI and elevated HbA1c levels are important predictors of poor treatment adherence. Another study involving children with T1D obtained similar findings: elevated HbA1c levels and low HRQoL (health-related quality of life) values were associated with increased prevalence of psychiatric comorbidity, affecting diabetes prognosis and treatment compliance [31]. Bratke et al. [32] obtained similar findings for elevated HbA1c levels in their comprehensive cross-sectional study involving children with T1D.

Various factors may influence this association in children with T1D; for instance, nutritional deficiencies, psychological factors, and the challenges surrounding insulin regulation in patients may contribute to issues with adherence to treatment regimens [18]. Lower BMI usually results from inadequate insulin replacement, which may predict non-adherence to diabetes management tasks (such as treatment adherence, blood glucose monitoring, and dietary intake) in children and adolescents with T1D. In addition, parents’ attitudes towards children’s nutrition, exercise, and diabetes management may significantly impact patients’ BMI, behavior, and treatment adherence, leading to less effective diabetes management in children [26].

According to the participants’ ASK-12 scores, 94 (58.8%) exhibited HTA, but 24 (25.5%) of them had poor metabolic control according to the pediatric endocrinologists’ assessment. This discordance between the physicians’ evaluation and the patients’ self-reports, as well as the low treatment adherence of approximately a quarter of the patients who were expected to be clinically compliant, is alarming. The presence of poor metabolic control may predispose patients to life-threatening clinical conditions, such as severe DKA and hypoglycemic convulsions. Identifying non-compliant patients and addressing factors affecting drug compliance are challenging, but these are essential tasks during the follow-up of patients with T1D. Similar findings were obtained by a multicentre study using the visual analogue scale and forced expiratory volume in one second (FEV1) to evaluate patients treated for asthma. More than half of the patients were non-compliant, inconsistent with the patients’ and physician’s assessments indicating high compliance [33].

A comprehensive meta-analysis emphasizes the importance of a multidisciplinary approach to improving medication adherence amongst children and adolescents, as well as the importance of an integrated approach to routine clinical care, with recurrent practice, assessment, and patient medication adherence recommendation [34]. Patients might be concerned about the negative impact that reporting treatment non-compliance might have on their communication with their clinicians [34]. The same physician followed-up on the majority of our participants. This attitude ensures continuity in chronic diseases and may improve patient-related compliance [35].

The ASK-12 questionnaire is a structured and comprehensible assessment tool for evaluating treatment adherence, with demonstrated internal consistency and reliability [10]. It includes questions that are easily understood by individuals across diverse age groups, making it practical for patients and their parents [17,36]. The ASK-12 questionnaire is brief and quick for routine implementation in outpatient clinics by healthcare providers, and it may help minimize the burden on patients. A semi-structured or self-administered questionnaire is a utility tool for identifying factors contributing to treatment non-adherence and facilitating targeted interventions to enhance patient compliance [10]. The ASK-12 scale facilitates a more comprehensive analysis of the underlying causes of non-adherence through items that assess patients’ perceived barriers, such as behavioural factors and knowledge about treatment [35]. This feature provides clinicians with insights to develop multifactorial intervention strategies. Additionally, the scale contributes to subjective and objective assessments, offering a more holistic evaluation of clinical decision-making [17]. It may also support an individualized approach to patient care by identifying barriers to optimal metabolic control and potentially reducing diabetic ketoacidosis admissions.

In our study, non-compliance was found in 25% of the participants who self-reported HTA, and this non-compliance was attributed to poor metabolic control as per the assessment of the pediatric endocrinologists. Similarly, Ekbom et al. [17], in their comprehensive study evaluating the ASK-12 questionnaire in patients with CAH, observed a discrepancy with the clinician’s assessment of one-third of the patients in the treatment-adherence group. A study found that approximately half of children with long-term medication for chronic diseases were non-adherent [37]. This finding demonstrates the challenge in recognizing patient-related clinical problems. In patients with T1D, treatment compliance and the reasons behind poor compliance should be investigated by clinicians at every check-up. The ASK-12 questionnaire is a practical tool for routine clinical use, providing a detailed assessment of the underlying factors influencing treatment adherence in pediatric T1D by evaluating patients’ perceived barriers. Additionally, it may aid in identifying the LTA and contributors to poor metabolic control. However, the HTA may also be associated with poor metabolic control. In children and adolescents with T1DM, patient self-management, parental support, sociocultural factors, and an individualized adherence-based follow-up approach are crucial for effective diabetes management.

None of the children in our cohort carried an emergency medical information identification card, although this is recommended by healthcare professionals. Concerns about being seen as different and concerns about exclusion are the possible reasons behind this observation. This is one issue that healthcare providers must address. The above findings highlight the significance of clinicians regularly addressing the barriers they observe in patients. Physicians’ simplified treatment regimens and comprehensible communication about treatment goals to develop strategies that would overcome resistance have helped improve medication adherence amongst pediatric patients requiring long-term treatment [37].

Notably, our center is one of the two primary diabetes centers in the city and also receives a significant number of referrals from neighboring provinces. We provide care for approximately half of the pediatric T1D patients, but our data may not entirely represent the widespread T1D population. Alassaf et al. [38]. found that children of mothers with higher education levels achieved better HbA1c outcomes, while financial constraints contributed to poor adherence and glycemic control. A study with Spanish children and adolescents with T1D found that those from nuclear families with higher socioeconomic status had better glycemic control, adherence, and quality of life [27]. Addressing socioeconomic conditions is crucial for improved diabetes management. Approximately 90% of our participants belonged to families with low socioeconomic status, and our study population was not homogeneous. One limitation of this study was that our participants were not evaluated by a psychiatrist. In this cross-sectional study, treatment compliance could have been evaluated using repeated interviews, as patients may not disclose all relevant information in a single meeting. Categorizing glycemic control as poor or tight glycemic control, using a qualitative variable, significantly limits the accuracy of the statistical analysis. Moreover, given that none of our patients used continuous glucose monitoring (CGM) systems, data on CGM parameters could not be evaluated. If CGM data were available, a more appropriate approach would be to consider quantitative variables (e.g., HbA1c, time in range, time in tight range, glycemic variability). However, we acknowledge that these measurements are subject to potential self-reported data, which would represent a limitation of the study.

Including subjects aged 5–18 years to assess treatment adherence would include significant variability in psychological maturity. A larger number of participants could help assess groups separately to determine variability in barriers by age period.

## 5. Conclusions

Routine screening of children and adolescents with T1D using the ASK-12 scale may reveal LTA and detect high-risk individuals for early diagnosis and intervention for barriers to diabetes management. Nearly a quarter of children with T1D exhibiting LTA based on their ASK-12 scores were found to be at risk of poor metabolic control. Examining the factors affecting metabolic control, entry to puberty, BMI, and parental involvement using the ASK-12 scale may help in managing patients’ treatment compliance.

## Figures and Tables

**Table 1:** Demographic and clinical findings of diabetes mellitus patients.

	Number (*n*)	Percentage (%)
Gender		
Female	96	60.00
Male	64	40.00
Puberty		
Non-pubertal	70	43.7
Presence of puberty	90	56.3
Remission		
Non-remitter	134	83.7
Partial remission	26	16.3
Diet Adherence		
Poor	82	51.2
Tight	78	48.8
Metabolic Control		
Poor	80	50.00
Tight	80	50.00

**Table 2:** Demographic and clinical findings of patients according to low treatment adherence and high treatment adherence.

	Low Treatment Adherence	High Treatment Adherence	*p*
	(*n* = 66)	(*n* = 94)	
Gender			
Female	45(68.2)	51(54.3)	0.198
Male	21 (31.8)	43 (45.7)	
Puberty	54 (81.8)	36 (38.3)	<0.001
Partial remission	2(3)	24 (25.5)	<0.001
Patient self-management	48 (72.7)	16 (17)	<0.001
Diet Adherence			
Poor	64 (97)	18 (19.1)	<0.001
Tight	2(3)	76(80.1)	
Metabolic Control			
Poor	66 (100)	24 (25.5)	<0.001
Tight	-	70 (74.5)	
	**Mean ± SD**	**Mean ± SD**	***p***
Age (years)	14.2 ± 2.3	9.9 ± 2.8	<0.001
Age of diagnosis	8.6 ± 2.5	7.7 ± 2.1	0.069
Duration of diabetes	5.4 ± 2.5	2.2 ± 2.2	<0.001
HbA1c (%)	11.4 ± 2.8	7.6 ± 0.9	<0.001
BMI-SDS	−0.47 ± 1.2	0.71 ± 0.71	<0.001
DKA event/year	2.15 ± 1.2	0.21 ± 0.6	<0.001

Body mass index standard deviation score: BMI-SDS. Diabetic ketoacidosis: DKA, mean ± standard deviation (SD).

## Data Availability

The datasets generated during and/or analyzed during the current study are available from the corresponding author on reasonable request due to privacy, legal, and ethical reasons.
